# Distinct parameters of the basophil activation test reflect the severity and threshold of allergic reactions to peanut

**DOI:** 10.1016/j.jaci.2014.09.001

**Published:** 2015-01

**Authors:** Alexandra F. Santos, George Du Toit, Abdel Douiri, Suzana Radulovic, Alick Stephens, Victor Turcanu, Gideon Lack

**Affiliations:** a Department of Pediatric Allergy, Division of Asthma, Allergy & Lung Biology, King's College London, London, United Kingdom; b MRC & Asthma UK Centre in Allergic Mechanisms of Asthma, London, United Kingdom; c Immunoallergology Department, Coimbra University Hospital, Coimbra, Portugal; d Gulbenkian Programme for Advanced Medical Education, Lisbon, Portugal; e Department of Public Health Science, School of Medicine, King's College London, London, United Kingdom; f National Institute for Health Research (NIHR), Biomedical Research Centre, Guy's and St Thomas' NHS Foundation Trust, London, United Kingdom

**Keywords:** Basophil activation test, peanut, peanut allergy, threshold, severity, sensitivity, CD63, CD203c, CD-sens, double-blind, placebo-controlled food challenge, CD63 peanut/anti-IgE, Ratio of the percentage of CD63^+^ basophils at 100 ng/mL of peanut extract to the percentage of CD63^+^ basophils after stimulation with anti-IgE, CD-sens, Basophil allergen threshold sensitivity, DBPCPC, Double-blind, placebo-controlled peanut challenge, EC_50_, Half maximal effective concentration, LEAP, Learning Early About Peanut Allergy, PA, Peanut allergy, PE, Peanut extract, SPT, Skin prick test

## Abstract

**Background:**

The management of peanut allergy relies on allergen avoidance and epinephrine autoinjector for rescue treatment in patients at risk of anaphylaxis. Biomarkers of severity and threshold of allergic reactions to peanut could significantly improve the care for patients with peanut allergy.

**Objective:**

We sought to assess the utility of the basophil activation test (BAT) to predict the severity and threshold of reactivity to peanut during oral food challenges (OFCs).

**Methods:**

The severity of the allergic reaction and the threshold dose during OFCs to peanut were determined. Skin prick tests, measurements of specific IgE to peanut and its components, and BATs to peanut were performed on the day of the challenge.

**Results:**

Of the 124 children submitted to OFCs to peanut, 52 (median age, 5 years) reacted with clinical symptoms that ranged from mild oral symptoms to anaphylaxis. Severe reactions occurred in 41% of cases, and 57% reacted to 0.1 g or less of peanut protein. The ratio of the percentage of CD63^+^ basophils after stimulation with peanut and after stimulation with anti-IgE (CD63 peanut/anti-IgE) was independently associated with severity (*P* = .001), whereas the basophil allergen threshold sensitivity CD-sens (1/EC_50_ × 100, where EC_50_ is half maximal effective concentration) value was independently associated with the threshold (*P* = .020) of allergic reactions to peanut during OFCs. Patients with CD63 peanut/anti-IgE levels of 1.3 or greater had an increased risk of severe reactions (relative risk, 3.4; 95% CI, 1.8-6.2). Patients with a CD-sens value of 84 or greater had an increased risk of reacting to 0.1 g or less of peanut protein (relative risk, 1.9; 95% CI, 1.3-2.8).

**Conclusions:**

Basophil reactivity is associated with severity and basophil sensitivity is associated with the threshold of allergic reactions to peanut. CD63 peanut/anti-IgE and CD-sens values can be used to estimate the severity and threshold of allergic reactions during OFCs.

Peanut allergy (PA) affects about 1.4% of children in the United States and 2% of children in the United Kingdom and is the most common cause of life-threatening anaphylaxis in childhood.^1,2^ The increase in hospitalization rates for peanut-induced anaphylaxis seems to follow the increase in the prevalence of PA.^3^ Patients often have a severe reaction on their first exposure to peanut.^4^ Oral food challenges (OFCs), the gold standard for diagnosis of PA, can also cause severe reactions in a significant proportion of cases.^5^ There is no curative treatment for PA, and the mainstay of its management is allergen avoidance and use of an epinephrine autoinjector as rescue treatment in severe cases. Allergen avoidance is difficult, and accidental allergic reactions are common.^6^ Patients with PA often react to small amounts of the allergens with symptoms that can be life-threatening, and thus PA has a negative effect on quality of life in patients and their families.

Current recommendations for the management of food allergy and anaphylaxis are based on expert opinion more than evidence-based randomized controlled trials.^7-9^ Various clinical factors have been identified as conferring a greater risk for severe food-induced allergic reactions, namely a previous history of anaphylaxis and the coexistence of uncontrolled asthma.^4^ However, most patients with fatal or near-fatal anaphylaxis had a history of mild allergic reactions, and not all had asthma.^10,11^ Therefore an objective biomarker that could accurately reflect the likelihood of experiencing severe allergic reactions for individual patients would be useful to help define indications for the prescription of an epinephrine autoinjector and help with risk assessment in patients who have to undergo an OFC for diagnostic purposes. Thus far, no reliable markers of severity or threshold have been identified. A recent article in this journal examined predictors of severity and threshold, including BATs in patients with PA, as confirmed based on challenge results.^12^ The authors found that no single parameter correlated with severity and that several parameters correlated with threshold doses.

Determining peanut threshold doses and whether a patient is likely to react to trace amounts of the allergen is an important aspect of the management of PA. Individual peanut thresholds could help define the stringency of allergen avoidance measures, and population peanut thresholds would be useful for public health authorities and the food industry to establish regulatory measures to protect patients with food allergy and institute allergen control measures and labeling policies. The current gold standard to determine threshold doses is a graded double-blind, placebo-controlled peanut challenge (DBPCPC) in patients known to have peanut allergy.^13^ This is logistically and technically demanding and carries significant risk; therefore an *ex vivo* method that could estimate threshold levels without the need for a DBPCPC would be very valuable.

We recently showed that the basophil activation test (BAT) reproduces very closely the phenotype of peanut-sensitized patients in relation to allergy versus tolerance.^14^ Basophils and mast cells are the effector cells of anaphylaxis. Basophils seem to be particularly relevant in patients with food-induced anaphylaxis, which often occurs without increased serum tryptase levels. Different methods to express the results of the BAT based on the allergen-induced dose-response curve reflect different aspects of the basophil response. The percentage of activated basophils measures basophil reactivity (eg, %CD63^+^ basophils at different allergen concentrations; the maximal %CD63^+^ basophils [maximal reactivity, CD-max] or the ratio of the %CD63^+^ after stimulation with allergen and with anti-IgE), and the concentration of allergen at which basophils become activated measures basophil sensitivity to the allergen (eg, EC5, EC10, EC50, CD-sens).^15^ We hypothesized that patients with severe reactions would show greater basophil reactivity and that patients who respond to lower doses of peanut allergen would show greater basophil sensitivity. We anticipated that higher percentages of activated basophils would result in higher percentages of basophils degranulating and higher amounts of vasoactive mediators released, leading to more severe symptoms. We anticipated also that the threshold dose for basophil activation and degranulation *in vitro* during the BAT would correspond to the threshold dose *in vivo* during the challenges.

## Methods

### Study population

Consecutive patients participating in a study about use of the BAT in the diagnosis of PA^14^ or in the Peanut Allergy and Sensitization study (which included children who had been excluded from the Learning Early About Peanut Allergy [LEAP] study^16^) with a positive oral peanut challenge result were included in this study. On the same day and before the challenge, all children underwent clinical evaluation, skin prick tests (SPTs), and blood collection for specific IgE determination and BATs. The study was approved by the South East London Research Ethics Committee 2. Written informed consent was obtained from the parents of all participants.

### SPTs and serum specific IgE measurement

SPTs were performed by using a commercially available peanut extract (PE; ALK-Abelló, Hørsholm, Denmark), as previously described.^14^ Serum specific IgE levels to peanut and to the recombinant peanut allergens rAra h 1, rAra h 2, rAra h 3, rAra h 8, and rAra h 9 were measured with an immunoenzymatic assay (ImmunoCAP; Thermo Fisher Scientific, Waltham, Mass).

### BAT

The BAT was performed, as previously described.^14^ Heparinized whole blood was stimulated for 30 minutes at 37°C with PE (ALK-Abelló) diluted in RPMI medium at serial 10-fold dilutions from 10 μg/mL to 0.1 ng/mL. Polyclonal goat anti-human IgE (Sigma-Aldrich, St Louis, Mo), monoclonal mouse anti-human FcεRI (eBioscience, San Diego, Calif), formyl-methionyl-leucyl-phenylalanine (Sigma-Aldrich), or RPMI alone were used as controls. Cells were stained with CD123–fluorescein isothiocyanate (eBioscience), CD203c-phycoerythrin, HLA-DR–peridinin-chlorophyll-protein complex, and CD63-allophycocyanin (BioLegend, San Diego, Calif), and erythrocytes were lysed. Basophils were gated as low side scatter/CD203c^+^/CD123^+^/HLA-DR^−^. Basophil expression of CD63 and CD203c was evaluated with the FACSCanto II with FACSDiva software (BD Biosciences, San Jose, Calif). Data were analyzed with FlowJo software, version 7.6.1 (TreeStar, Ashland, Ore).

### Oral peanut challenges and determination of severity and threshold

Six verum doses and 3 placebo doses were randomly interspersed with verum doses up to a cumulative dose of 9.35 g of peanut protein (see Table E1 in this article's Online Repository at www.jacionline.org). Children 1 to 3 years of age were given 5 verum doses and 1 placebo dose up to a cumulative dose of 4.35 g of peanut protein. High-risk patients (ie, patients with suspected PA, a history of life-threatening food-induced anaphylaxis, or an SPT response ≥7 mm) received an additional lower starting active dose of 0.033 g of peanut protein. DBPCPCs were performed in 92% of cases. Four patients had open challenges for logistic reasons, as previously reported.^14^ The challenge result was considered positive only when objective signs of an allergic reaction developed (see Table E2 in this article's Online Repository at www.jacionline.org) and the symptoms were treated. In the case of a reaction after a placebo dose, the OFC was repeated with a 2-day protocol (1 day of placebo and 1 day of verum); otherwise, OFCs were performed in a single day.^17^

Allergic reactions to peanut during challenges were attributed a symptom score varying between 1 and 5, and the severity was classified into mild, moderate, or severe categories by using a published method.^18^ Patients were dichotomized depending on whether their reaction was mild-moderate or severe (see Table E3 in this article's Online Repository at www.jacionline.org). Severity was classified by using 4 additional severity scores.^19-22^

The threshold dose for OFCs was defined as the cumulative threshold dose of peanut protein at the time of the reaction as opposed to a discrete threshold dose. Patients were grouped according to the cumulative threshold dose at the time of reaction into low (≤0.1 g of peanut protein) versus high (>0.1 g of peanut protein) threshold doses. Discrete threshold doses (ie, the dose administered immediately before the positive response) were also recorded.^23^

### Statistical analysis

We predicted that severity would be related to basophil reactivity and that threshold would be related to basophil sensitivity. We decided to look primarily at the proportion of activated basophils at different peanut concentrations and CD63 peanut/anti-IgE for severity and at half maximal effective concentration (EC_50_) and basophil allergen threshold sensitivity (CD-sens) values for threshold and also at other markers, such as the area under the dose-response curve.^15^

Qualitative variables were represented as proportions and compared between severity or threshold groups by using the Fisher exact test. Continuous variables were represented as medians and interquartile ranges and compared with the Mann-Whitney *U* test.

Allergy test parameters noted to have differences (*P* < .1) between groups were further tested as independent variables in logistic regression analyses by using the severity- or threshold-dichotomized groups as dependent variables. The best performing BAT parameter (based on the lowest *P* value) was tested in the logistic regression analysis: for severity, this parameter was the ratio of the percentage of CD63^+^ basophils at 100 ng/mL of PE to the percentage of CD63^+^ basophils after stimulation with anti-IgE (CD63 peanut/anti-IgE), and for threshold, this parameter was CD-sens, which was determined by using CD63 (CD-sens is the inverse of the half-maximal effective concentration [ie, the concentration at which basophil activation is half of the maximum activation] times 100 and can be calculated by using the following formula: CD-sens = 1/EC_50_ × 100), as previously described by Johansson et al.^24^ Independent associations between severity or threshold and allergy test parameters were further investigated by using multivariable logistic regression analyses, and only variables significantly contributing to the model (*P* < .05) were retained.

Patients were dichotomized based on the 75th percentile of CD63 peanut/anti-IgE or CD-sens values and the proportion of patients with severe reactions or low thresholds were compared between those falling above and below the 75th percentile to quantify the differences in basophil activation between severity and threshold groups. BAT cutoffs for severity and threshold were also determined by using receiver operating characteristic curve analyses.

The correlation between the clinical and diagnostic test parameters for severity and threshold were assessed by using Spearman correlation.

Statistical analyses were performed with SPSS 22.0 software for Windows (SPSS, Chicago, Ill) and MedCalc 13.3 (MedCalc Software, Ostend, Belgium). *P* values of less than .05 were considered statistically significant.

## Results

### Study population

One hundred twenty-four patients were submitted to oral peanut challenges. Fifty-two (42%) patients had a positive challenge result to peanut. Three patients showed nonresponder basophils and were excluded from further analyses. The study population (n = 49, Table I) was aged from 1.6 to 13 years (median age, 5 years), and the majority (77%) had never ingested peanut before the challenge.

### Severity of allergic reactions during peanut challenges

Symptoms during the challenge ranged from mild oral symptoms to anaphylaxis. Twenty (41%) patients had severe reactions (see Table E4 in this article's Online Repository at www.jacionline.org). Nine (18%) patients required the administration of intramuscular epinephrine, and 10 (77%) required the administration of intravenous fluid boluses. One patient had a biphasic reaction about 5 hours after the resolution of the allergic symptoms that occurred during the challenge.

Severe reactors had comparable SPT responses (*P* = .102) and higher levels of specific IgE to peanut (*P* = .010), Ara h 1 (*P* = .021), and Ara h 2 (*P* = .003) compared with the patients who had mild-to-moderate reactions. Having a greater number of peanut major allergens (Ara h 1, Ara h 2, and Ara h 3) recognized by patients' IgE was also associated with severe reactions (*P* = .019). Patients who received intramuscular epinephrine had higher specific IgE levels to peanut (*P* = .031) and Ara h 2 (*P* = .011) than patients who did not require epinephrine (see Table E4).

### Threshold of allergic reactions during peanut challenges

The cumulative threshold dose of peanut protein varied between 0.033 and 9.35 g (median, 0.1 g). Twenty-eight (57%) patients reacted to 0.1 g or less of peanut protein during the OFCs (see Table E4) and had larger wheals on SPTs to peanut (*P* = .021) and higher levels of specific IgE to peanut (*P* = .026) and Ara h 2 (*P* = .032) than patients who reacted to more than 0.1 g of peanut protein (Table I). Interestingly, patients with a higher cumulative peanut threshold dose had a higher ratio of peanut-specific IgG_4_ to IgE (*P* = .011). Classifying patients according to the discrete threshold dose of peanut protein at the time of the reaction yielded similar findings (data not shown).

### Severity of allergic reactions to peanut is associated with greater allergen-specific basophil reactivity

**Fig 1:**
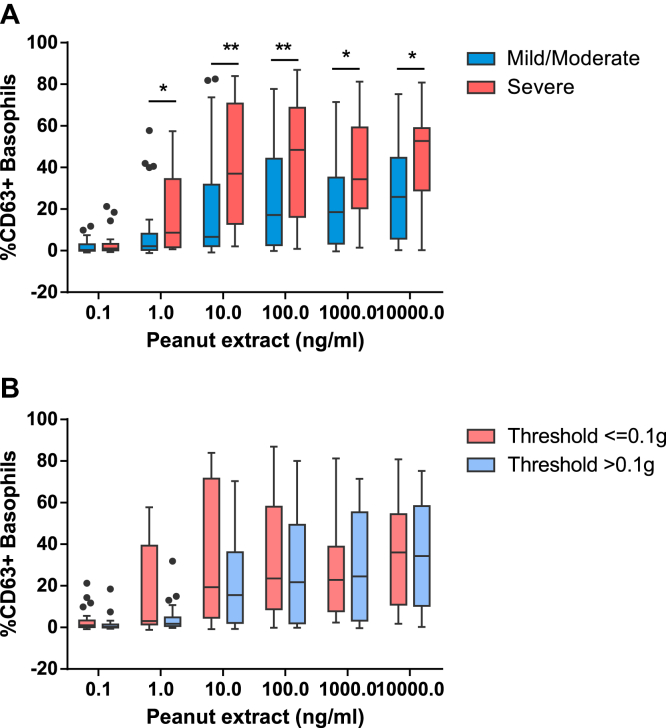
Peanut dose response of basophil activation in patients with severe versus nonsevere reactions **(A)** and in patients with low versus high threshold of reactivity **(B)** to peanut. *P < .05 and **P < .01 for the comparison between groups by using the Mann-Whitney *U* test.

**Fig 2:**
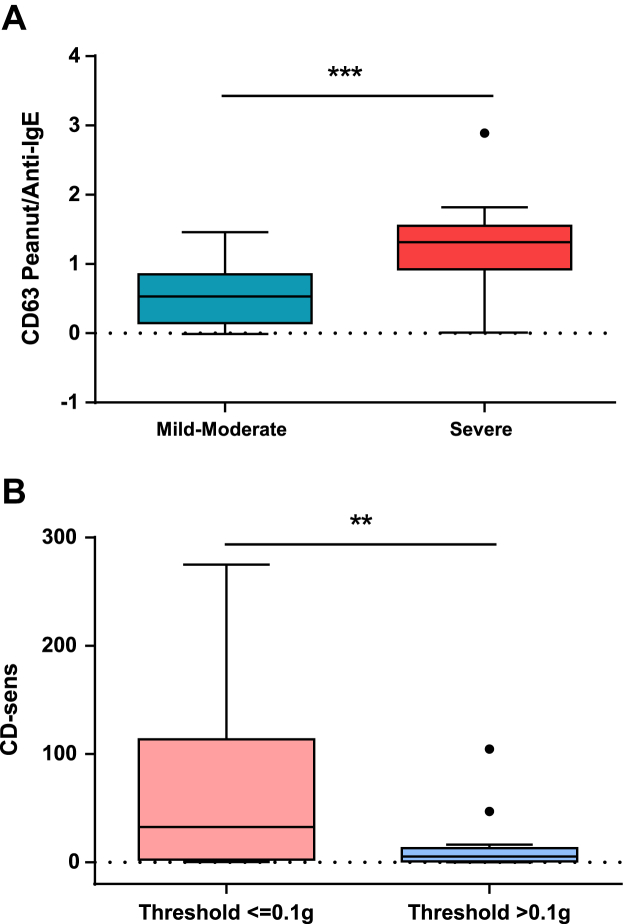
Best BAT parameters to distinguish between patients with severe versus nonsevere reactions **(A)** and patients with low versus high threshold of reactivity **(B)** to peanut. **P < .01 and ***P < .001 for the comparison between groups by using the Mann-Whitney *U* test.

Patients with severe reactions to peanut during OFCs showed a higher proportion of CD63^+^ basophils at concentrations of PE ranging from 1 to 10,000 ng/mL compared with patients with PA with mild-to-moderate reactions (*P* = .003-.049; Fig 1, *A*, and Table II). The best basophil markers for the severity of allergic reactions, based on the lowest *P* value, were the ratio of the percentage of CD63^+^ basophils at 100 ng/mL peanut to the percentage of CD63^+^ basophils after stimulation with anti-IgE (CD63 peanut/anti-IgE, *P* < .001; Fig 2, *A*, and Table II) and the percentage of CD63^+^ basophils at 100 ng/mL PE (*P* = .003). The latter marker was previously identified as optimal for the diagnosis of PA^14^ and was the best discriminator of the patients who had severe reactions requiring the administration of intramuscular epinephrine (see Table E5 in this article's Online Repository at www.jacionline.org).

### Basophil sensitivity indicates the threshold of allergic reactions to peanut

The dose response for peanut-induced basophil activation of patients with lower cumulative peanut threshold doses on OFCs was shifted to the left compared with the dose response of patients with a higher cumulative peanut threshold dose (Fig 1, *B*). Patients with PA with lower threshold doses showed higher basophil sensitivity, as expressed by a higher CD-sens value (*P* = .005) and a correspondingly lower EC_50_ value (*P* = .019; Fig 2, *B*, and Table II). Similar differences were found when considering discrete threshold doses (data not shown).

### Logistic regression analyses to assess different parameters of severity and threshold

Because parameters other than BAT varied with the severity and threshold of allergic reactions to peanut (Table I), logistic regression analyses were conducted to assess which parameters were independently associated with severity and threshold (Table III). After multivariable analyses, only the basophil activation markers were retained for severity and threshold, meaning that the BAT alone was more discriminative in predicting the severity (CD63 peanut/anti-IgE, *P* = .001) and threshold (CD-sens, *P* = .020) of allergic reactions to peanut than the other allergy tests or the combination of the BAT with the other allergy tests (Table III). Classifying the severity of allergic reactions according to the other severity scores and according to the need for epinephrine resulted in similar findings (data not shown).

### Basophil activation test cutoffs for severity and threshold

**Fig 3:**
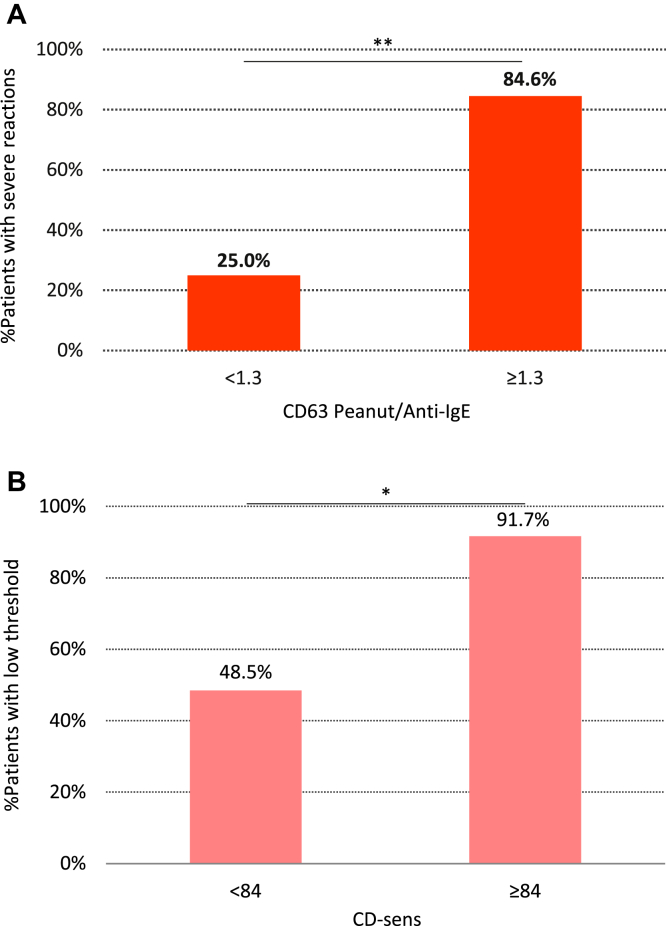
**A,** Proportion of patients with severe reactions according to the 75th percentile of basophil reactivity (measured by CD63 peanut/anti-IgE). **B,** Proportion of patients with lower threshold of reactivity to peanut according to the 75th percentile of basophil sensitivity (measured by using CD-sens). *P = .05 and **P < .01 for the comparison between groups by using the Mann-Whitney *U* test.

Basophil activation of 1.3 or greater CD63 peanut/anti-IgE increased the proportion of severe reactors by 3-fold (relative risk, 3.4; 95% CI, 1.8-6.2) compared with that seen in patients with less than 1.3 CD63 peanut/anti-IgE (*P* = .001; Fig 3, *A*). Patients with CD-sens values of 84.0 or greater had an about 2-fold higher chance of reacting to trace amounts of peanut (relative risk, 1.9; 95% CI, 1.3-2.8) compared with patients with lower CD-sens values and thus with a higher threshold of reactivity to peanut (*P* = .014). BAT result cutoffs to estimate the severity and threshold of peanut-induced allergic reactions were also determined by using receiver operating characteristic curve analyses (see Fig E1 in this article's Online Repository at www.jacionline.org).

### Correlation between severity and threshold

Clinically, symptom score and threshold dose were not correlated (*Rs* = −0.067, *P* = .645, see Table E6 in this article's Online Repository at www.jacionline.org). However, the basophil markers of severity (CD63 peanut/anti-IgE) and threshold (CD-sens) were strongly correlated (*Rs* = 0.60, *P* < .001).

## Discussion

Current management of PA relies on allergen avoidance and the prescription of autoinjectable epinephrine to patients deemed to be at risk of anaphylaxis. Knowing whether individual patients are at risk of reacting to trace amounts of the allergen or of having severe reactions would improve the care for patients with PA. Previously, we showed that the BAT resembles very closely the clinical phenotype of patients in terms of clinical reactivity to peanut.^14^ In this study we identified allergen-specific basophil reactivity (as measured by CD63 peanut/anti-IgE) and basophil sensitivity (as measured by CD-sens) as biomarkers of severity and threshold of allergic reactions to peanut during OFCs.

This is a prospective study of a well-characterized population of patients with PA that were submitted to OFCs regardless of the presence of clinical risk factors for severe reactions and of the SPT and specific IgE results. In most previous studies patients with a previous history of anaphylaxis or current asthma and/or with specific IgE levels of greater than the 95% positive predictive value cutoff were often excluded, thereby limiting the spectrum of the disease severity studied.^25^ Different severity scores have been adopted in different studies, some including both symptom score and eliciting dose. We adopted a severity score that was previously validated^18,26^ and does not include the dose that caused a reaction because we aimed to assess these 2 factors, severity and threshold, independently. Indeed, distinct BAT parameters reflected the severity and threshold of allergic reactions. The best parameter to predict severity was the ratio between basophil-specific activation to allergen and basophil-nonspecific activation to anti-IgE. The response of basophils of allergic patients to allergen has been reported to be greater than that to anti-IgE or anti-FcεRI.^27,28^ In a previous study of children with cow's milk allergy,^28^ the ratio between the percentage of CD63 basophils in response to cow's milk and to anti-FcεRI was higher in patients with persistent cow's milk allergy compared with that in patients who outgrew their allergy and was correlated with the severity of the reactions during challenges.

In contrast to a study recently published in the *Journal*,^12^ in which BAT results showed no correlation with severity but only with threshold, in our study BATs informed not only about threshold but also about the severity of allergic reactions during OFCs. Although different severity scores were primarily used, in both studies findings were confirmed with other severity scores; thus the severity scores used were unlikely to have accounted for the discrepancy between results. These differences might be explained by the adopted OFC protocol. In the cited study OFCs were performed over 2 days, with 2-hour intervals between doses up to a cumulative dose of 4.443 g of peanut protein. In our study OFCs were performed on a single day with 20-minute intervals between doses, and the cumulative dose for children older than 3 years was 9.35 g. These factors could have contributed to the greater severity of reactions we observed during OFCs, enabling us to find a biomarker for severity. We also found stronger correlations for threshold and additionally performed logistic regression analyses and determined cutoffs both for severity and threshold.

It should be noted in our study that a single-day challenge protocol is performed in which placebo and active doses are interspersed. This is not in keeping with the recent PRACTALL guidelines^29^ but is in keeping with the LEAP study–recommended challenge procedure to peanut (NCT00329784).^16,30^ This is done for pragmatic reasons because a large number of children come from further afield in the United Kingdom, and it is difficult for them to come in for a 2-day OFC program. On the rare occasion that a child reacts after a placebo dose, then a 2-day DBPCFC is performed in which placebo doses are administered on one day and active doses on the other day. Only 2 of 52 patients with positive challenge results in the study initially reacted after placebo on a mixed active-placebo 1-day protocol, and both these patients had their diagnosis of PA subsequently confirmed by using a 2-day DBPCFC.

With respect to SPT responses and serum specific IgE levels to peanut possibly reflecting the severity of allergic reactions to peanut, previous studies showed contradictory results, with some showing that SPT responses and serum specific IgE levels predicted the development of anaphylaxis^6,31-33^ and others not finding any association between allergy test results and the severity of allergic reactions.^4,25^ In our study, patients with severe reactions had higher levels of specific IgE to peanut, Ara h 1, and Ara h 2. Severity has also been associated with a greater number of peanut allergens^34,35^ and epitopes^36^ recognized by patients' IgE and with the intensity of bands on immunoblotting as a surrogate for antibody affinity and avidity.^34^ Our study corroborates these findings because patients with severe reactions had IgE directed to a larger number of peanut major allergens compared with patients with mild-to-moderate reactions. Interestingly, patients with a higher ratio of peanut-specific IgG_4_ to IgE reacted to higher doses of peanut, suggesting that IgG_4_ competed with IgE for binding to the allergen, blocking its effect and preventing degranulation of basophils at a low PE concentration. One of the advantages of the BAT is that it is a functional assay that takes into account all these factors, including levels, specificity, diversity, and affinity of allergen-specific IgE and even possible interference by other allergen-specific antibodies, which together are responsible for allergen-induced effector cell activation. Therefore the BAT has a greater potential to reflect the allergic reaction as it happens *in vivo* than methods that test these IgE parameters separately. This is reflected in our data in which, after multivariable analyses, BAT parameters proved to be more predictive of severity and threshold of allergic reactions than the other tests.

A relationship between severity and threshold has previously been suggested,^37^ with patients who react to lower doses being more at risk of severe symptoms. In our study and others^12,38^ the clinical parameters of severity and threshold were not correlated. However, a strong correlation was found for the respective basophil parameters. This discrepancy could be due to the fact that during the BAT, the “*in vitro* challenge” can progress to higher doses, whereas the *in vivo* OFC is typically stopped with the first allergic symptoms and signs. Allergic reactions during OFCs might have been different if a large dose had been consumed at once. The fact that basophil activation can be determined up to high doses of allergen regardless of disease severity is another clear advantage of the BAT as a biomarker of disease severity.

We identified BAT markers of severity and threshold of reactions during OFCs, but they might not reflect the severity and threshold of allergic reactions in the community. Hourihane et al^33^ showed that the challenge score correlated with the most recent reaction but not with the most severe reaction in the community, suggesting that a patient's reactivity to peanut changes over time. Cofactors that can increase the severity of allergic reactions (eg, uncontrolled asthma, viral infections, menstruation, exercise, consumption of alcohol or drugs, and psychological factors) might be present in the community and are usually controlled during OFCs. Also, with respect to threshold, there might be some variation because the eliciting dose during OFCs can be underestimated because the patient feels safe during the OFC but can also be overestimated because mild symptoms would remain unnoticed in the community.^22^ Severity and eliciting dose during challenges have not been reproducible in other studies.^39,40^

The utility of the BAT as a marker for severity and threshold of allergic reactions is to provide additional information to the patients and should be interpreted in light of the clinical history and presence of other risk factors. The management of patients should continue to be based on patient education, and the importance of an emergency treatment plan and appropriate training cannot be overemphasized, regardless of the magnitude of allergy test results, including those of the BAT. However, the BAT can be used to identify patients who are at risk of reacting to small amounts of the allergen and of having severe symptoms that require special attention. Further validation of these objective BAT markers in different populations repeated at different time points might allow us to identify the subset of high-risk children with PA who require closer monitoring, as well as the subgroup of children whose allergy to peanut might spontaneously resolve. Identification of high-risk groups should not be based only on biological markers but also on psychosocial, demographic, behavioral, and clinical parameters. Future studies on the stability of BAT results over time and during different periods of the year (eg, hay fever season and asthma exacerbations) would be most informative. Although BAT parameters might not completely distinguish between those patients with severe reactions who respond to a low threshold of reactivity and those with milder reactions and a higher threshold dose, they could prove to be far more accurate in discriminating changes in the clinical threshold and severity in the same subjects over time. It has already been shown that BAT results to peanut, egg, and milk decrease in patients who have undergone oral immunotherapy.^41-43^ It would be of great value to look at the stability of BAT results over time in untreated patients and at the change in BAT parameters in patients undergoing oral immunotherapy to foods and compare this with posttreatment challenge outcome measures.

**Clinical implications**

The BAT can be used as an *in vitro* surrogate for OFCs to estimate the severity and threshold of allergic reactions and improve the management of patients with PA.

## Figures and Tables

**Table I:** Characteristics of the study population according to severity and threshold groups

Demographic features and investigations	Study population (n = 49)	Severity groups			Threshold groups		
		Mild-moderate (n = 29)	Severe (n = 20)	*P* value	≤0.1 g Peanut protein (n = 28)	>0.1 g Peanut protein (n = 21)	*P* value
Age (y)	5.4 (4.7-5.9)	5.2 (4.4-5.8)	5.4 (5.0-6.2)	.250	5.3 (4.8-5.8)	5.4 (4.6-6.1)	.928
Male sex, no. (%)	34 (69.4)	19 (65.5)	15 (75.0)	.542	18 (64.3)	16 (76.2)	.533
Symptom score	3 (3-4)	3 (3-3)	4 (4-5)	∗	3 (3-4)	3 (3-4)	.501
Cumulative threshold dose of peanut protein (g)	0.1 (0.03-0.63)	0.10 (0.33-1.38)	0.10 (0.03-0.38)	.884	0.03 (0.03-0.09)	0.92 (0.37-4.38)	∗
SPT response to peanut (mm)	9 (7-12)	9 (4-10)	10 (7-13)	.102	10 (8-13)	8 (4-11)	**.021**
Specific IgE to peanut (KU_A_/L)	5.15 (0.51-29.78)	1.86 (0.38-12.85)	23.20 (5.18-94.80)	**.010**	7.17 (1.73-75.80)	1.33 (0.38-12.35)	**.026**
Specific IgE to Ara h 1 (KU_A_/L)	0.11 (0.02-2.14)	0.09 (0.01-0.23)	0.20 (0.09-11.58)	**.021**	0.18 (0.04-8.67)	0.09 (0.01-0.15)	.051
Specific IgE to Ara h 2 (KU_A_/L)	1.65 (0.20-15.20)	0.68 (0.10-2.67)	10.11 (1.48-41.40)	**.003**	5.05 (1.06-46.80)	0.46 (0.12-7.36)	**.032**
Specific IgE to Ara h 3 (KU_A_/L)	0.03 (0.01-0.33)	0.03 (0.01-0.13)	0.05 (0.01-0.98)	.221	0.04 (0.01-0.89)	0.02 (0.01-0.05)	.069
Specific IgE to Ara h 8 (KU_A_/L)	0.03 (0.01-0.47)	0.03 (0.01-0.23)	0.03 (0.01-1.96)	.582	0.03 (0.01-0.28)	0.02 (0.01-2.04)	1.0
Specific IgE to Ara h 9 (KU_A_/L)	0.01 (0.01-0.02)	0.01 (0.01-0.02)	0.01 (0.01-0.07)	.818	0.01 (0.01-0.04)	0.01 (0.01-0.02)	.856
No. of major peanut allergens bound by IgE	2 (1-2)	1.0 (0-2.0)	2.0 (1.0-3.0)	**.019**	2 (1-3)	1 (1-2)	.089
Peanut-specific IgG_4_ (μg/L)	190.0 (120.0-662.5)	160 (120-405)	380 (120-1100)	.235	0.14 (0.12-0.80)	0.29 (0.13-0.66)	.425
Ratio of peanut-specific IgG_4_ to IgE	15.7 (5.7-88.3)	21.63 (5.78-161.23)	11.26 (3.15-76.75)	.209	8.6 (2.3-66.1)	48.9 (15.5-159.4)	**.011**
Other food allergy, no. (%)	47 (95.9)	27 (93.1)	20 (100.0)	.507	28 (100.0)	19 (90.5)	.179
Atopic eczema, no. (%)	40 (81.6)	23 (79.3)	17 (85.0)	.720	25 (89.3)	15 (71.4)	.146
Asthma, no. (%)	19 (38.8)	11 (37.9)	8 (40.0)	1.0	11 (39.3)	8 (38.1)	1.0
Allergic rhinitis, no. (%)	27 (55.1)	15 (51.7)	12 (60.0)	.771	19 (67.9)	8 (38.1)	**.048**
Pollen allergy, no. (%)	14 (28.6)	7 (24.1)	7 (35.0)	.524	8 (28.6)	6 (28.6)	1.0

Values are expressed as numbers (percentages) or medians (interquartile ranges). *P* values < .05 are boldface.

∗ Values are not indicated because these characteristics formed the basis for classifying the patients into severity and threshold groups (*P* <. 001).

**Table II:** BAT parameters associated with the severity and threshold of allergic reactions to peanut

BAT to peanut	Study population (n = 49)	Severity groups			Threshold groups		
		Mild-moderate (n = 29)	Severe (n = 20)	*P* value	≤0.1 g Peanut protein (n = 28)	>0.1 g Peanut protein (n = 21)	*P* value
% CD63^+^ peanut 0.1	0.63 (0-3.05)	0.36 (0-3.05)	1.03 (0.16-3.22)	.215	1.07 (0.14-3.34)	0.36 (0-1.43)	.140
% CD63^+^ peanut 1	2.38 (0.90-15.44)	2.13 (0.36-8.12)	8.60 (1.68-34.41)	**.016**	3.08 (1.41-39.38)	1.76 (0.68-4.85)	.090
% CD63^+^ peanut 10	18.16 (4.45-51.90)	6.65 (2.15-31.73)	37.04 (12.80-70.71)	**.009**	19.33 (4.67-71.52)	15.54 (2.15-36.12)	.163
% CD63^+^ peanut 100	21.78 (8.44-51.88)	17.11 (2.60-44.28)	48.44 (16.24-68.79)	**.003**	23.60 (8.73-58.03)	21.78 (1.99-49.36)	.505
% CD63^+^ peanut 1,000	23.53 (7.70-43.37)	18.56 (3.37-35.18)	34.33 (20.31-59.27)	**.049**	22.86 (7.70-38.88)	24.57 (3.30-55.36)	.818
% CD63^+^ peanut 10,000	34.36 (12.32-55.44)	25.86 (5.74-44.68)	52.79 (28.92-58.93)	**.012**	36.08 (10.96-54.47)	34.36 (10.40-58.37)	.888
Mean % CD63 peanut 10-100	16.94 (6.29-52.38)	13.26 (2.56-38.05)	41.31 (10.23-67.36)	**.012**	10.18 (5.16-16.09)	20.41 (4.36-43.42)	.419
AUC CD63 peanut	86.96 (46.82-207.38)	66.44 (27.64-164.91)	159.14 (78.84-240.42)	**.016**	92.96 (34.24-231.34)	86.96 (52.84-196.60)	.671
Maximal % CD63^+^ to peanut	39.90 (15.20-67.39)	27.74 (9.99-56.23)	59.49 (31.47-75.85)	**.025**	47.59 (15.12-74.75)	34.66 (13.08-62.81)	.303
% CD63^+^ peanut/anti-IgE	0.82 (0.32-1.32)	0.53 (0.15-0.85)	1.32 (0.92-1.55)	**<.001**	0.79 (0.44-1.26)	0.83 (0.09-1.35)	.716
EC_50_ (ng/mL)	10 (1-10)	10 (1-100)	10 (1-10)	.058	1.0 (1.0-32.5)	10.0 (10.0-10.0)	**.019**
CD-sens	12.97 (1.96-85.91)	5.37 (0.99-50.66)	32.59 (11.62-87.97)	**.023**	32.59 (2.58-113.75)	5.37 (0.80-13.13)	**.005**

Values are expressed as medians (interquartile ranges). Peanut extract concentrations are expressed in ng/mL. EC_50_ and CD-sens were calculated using the CD63 dose-response. *P* values < .05 are boldface. *AUC*, Area under the curve.

**Table III:** Univariable and multivariable analyses of factors associated with the severity and threshold of allergic reactions to peanut

Variable	Severe allergic reaction		Low threshold of reactivity	
	OR (95% CI)	*P* value	OR (95% CI)	*P* value
Univariable analysis				
% CD63^+^ peanut/anti-IgE	0.111 (0.026-0.478)	**.001**	—	—
CD-sens	—	—	1.027 (1.004-1.050)	.020
SPT	—	—	1.231 (1.033-1.466)	.020
Specific IgE to peanut	1.014 (1.0-1.029)	.056	1.022 (1.0-1.045)	.053
Specific IgE to Ara h 1	1.031 (0.985-1.079)	.186	1.016 (0.975-1.058)	.459
Specific IgE to Ara h 2	1.034 (0.999-1.070)	.054	1.036 (0.994-1.080)	.090
Specific IgE to Ara h 3	—	—	3.391 (0.590-19.501)	.171
No. of major peanut allergens bound by IgE	2.342 (1.144-4.791)	.020	1.712 (0.896-3.272)	.104
Peanut-specific IgG_4_/IgE ratio	—	—	0.999 (0.997-1.001)	.283
Allergic rhinitis	—	—	3.431 (1.049-11.222)	.041
Multivariable analysis∗				
% CD63^+^ peanut/anti-IgE	0.111 (0.026-0.478)	**.001**	—	—
CD-sens	—	—	1.027 (1.004-1.050)	**.020**

∗ All variables were retested by using forward multivariable logistic regression, and only variables contributing to the model (*P* < .05) were retained. CD-sens refers to CD-sens values calculated by using the CD63 dose response. *P* values < .05 are boldface.

## Supplementary data

Online Repository Data

Figure E1

Figure E2
