# Supplementary material for: Distinct parameters of the basophil activation test reflect the severity and threshold of allergic reactions to peanut
Source: J Allergy Clin Immunol. 2015 Jan;135(1):179–86. doi: 10.1016/j.jaci.2014.09.001 (PMC4282725; doi:10.1016/j.jaci.2014.09.001)
Supplement: Online Repository Data [file mmc1.docx]

**Online Repository Material:**

**Table E1.** Doses of peanut protein in the oral food challenge protocol. Placebo doses were randomly interspersed with verum doses. *Additional starting dose for high-risk patients.

| **DOSES** | **Peanut protein (g)** |
| --- | --- |
| 1* | 0.033* |
| 2 | 0.1 |
| 3 | 0.25 |
| 4 | 0.5 |
| 5 | 1.0 |
| 6 | 2.5 |
| 7 | 5.0 |

**Table E2.** Criteria for positive oral food challenge to peanut.

| **Major criteria** | Confluent erythematous pruritic rash |
| --- | --- |
|  | Wheezing |
|  | Stridor |
|  | Dysphonia / Aphonia |
|  | ≥ 3 urticarial lesions |
|  | ≥ 1 site of angioedema |
|  | Hypotension for age not related to vasovagal episode |
|  | Evidence of severe abdominal pain that persists for ≥3 minutes |
| **Minor criteria** | Vomiting |
|  | Diarrhoea |
|  | Persistent rubbing of eyes that last ≥3 minutes |
|  | Persistent rhinorrhea that lasts ≥3 minutes |
|  | Persistent scratching that lasts ≥3 minutes |

Footnote: A positive oral food challenge (OFC) was defined by the presence of either ≥1 major criteria or ≥2 minor criteria. An indeterminate OFC was defined as one minor criterion. A negative OFC was defined by the absence of major or minor criteria.

**Table E3.** Classification of allergic reactions to peanut according to severity^1^.

| **Symptom score** | | **Severity classification** |
| --- | --- | --- |
| **1** | Localized cutaneous erythema  Localized urticaria  Localized angioedema  Oral pruritus | **Mild** |
| **2** | Generalized erythema  Generalized urticaria  Generalized angioedema |  |
| **3** | Gastrointestinal symptoms  Rhinitis | **Moderate** |
| **4** | Laryngeal edema  Mild asthma | **Severe** |
| **5** | Dyspnea  Hypotension |  |

**Table E4.** Severity and threshold of allergic reactions to peanut during the challenges (n=49).

| **Symptom score** | **Number (%)**  **of patients** | **Cumulative threshold dose of peanut protein (g)** | **Number (%)**  **of patients** |
| --- | --- | --- | --- |
| 1 | 4 (8.2%) | 0.033 | 19 (39%) |
| 2 | 2 (4.1%) | >0.033 <0.1 | 2 (4%) |
| 3 | 23 (46.9%) | 0.1 | 7 (14%) |
| 4 | 15 (30.6%) | >0.1 <4 | 14 (29%) |
| 5 | 5 (10.2%) | ≥4 | 7 (14%) |

**Table E5.** Demographic and clinical characteristics and BAT parameters according to requirement of intramuscular epinephrine.

| **Demographic and clinical features** | **Epinephrine required (n=9)** | **Epinephrine not required (n=40)** | **p value** |
| --- | --- | --- | --- |
| Age (years) | 5.15 (4.94; 6.31) | 5.39 (4.63; 5.85) | 0.786 |
| Males - n (%) | 6 (66.7%) | 28 (70.0%) | 1.0 |
| Cumulative threshold dose of peanut protein | 0.10 (0.03; 0.24) | 0.10 (0.03; 1.62) | 0.579 |
| SPT to peanut (mm) | 10 (8; 16) | 9 (5; 11) | 0.111 |
| Specific IgE to peanut (KU_A_/L) | 27.50 (15.93; 87.65) | 2.74 (0.47; 16.53) | **0.031** |
| Specific IgE to Ara h 1 (KU_A_/L) | 0.13 (0.01; 3.25) | 0.10 (0.02; 0.41) | 0.667 |
| Specific IgE to Ara h 2 (KU_A_/L) | 15.20 (9.21; 54.30) | 1.09 (0.17; 6.60) | **0.011** |
| Specific IgE to Ara h 3 (KU_A_/L) | 0.05 (0.01; 1.10) | 0.03 (0.01; 0.24) | 0.333 |
| Specific IgE to Ara h 8 (KU_A_/L) | 0.03 (0.02; 2.04) | 0.02 (0.01; 0.28) | 0.339 |
| Specific IgE to Ara h 9 (KU_A_/L) | 0.02 (0.01; 0.04) | 0.01 (0.01; 0.02) | 0.653 |
| Number of major peanut allergens bound by IgE | 2 (1; 3) | 2 (1; 2) | 0.309 |
| Peanut-specific IgG4 (µg/L) | 590 (180; 1043) | 160 (120; 575) | 0.135 |
| Ratio of peanut-specific IgG4 to IgE | 10.05 (1.90; 149.91) | 17.62 (5.88; 88.33) | 0.346 |
| Other food allergy - n (%) | 9 (100.0%) | 38 (95.0%) | 1.0 |
| Atopic eczema - n (%) | 7 (77.8%) | 33 (82.5%) | 0.663 |
| Asthma - n (%) | 3 (33.3%) | 16 (40.0%) | 1.0 |
| Allergic rhinitis - n (%) | 7 (77.8%) | 20 (50.0%) | 0.159 |
| Pollen allergy - n (%) | 4 (44.4%) | 10 (25.0%) | 0.254 |
| **BAT parameters** | **Epinephrine required (n=9)** | **Epinephrine not required (n=40)** | **p value** |
| %CD63+ Peanut 0.1 | 3.31 (1.98; 12.96) | 0.33 (0; 1.18) | **0.001** |
| %CD63+ Peanut 1 | 5.65 (8.60; 45.65) | 1.95 (0.67; 9.42) | **0.001** |
| %CD63+ Peanut 10 | 59.63 (37.04; 73.93) | 10.69 (2.46; 35.16) | **0.002** |
| %CD63+ Peanut 100 | 68.01 (45.09; 74.66) | 17.59 (6.08; 46.41) | **<0.001** |
| %CD63+ Peanut 1,000 | 49.65 (35.13; 64.49) | 20.31 (3.54; 34.33) | **0.002** |
| %CD63+ Peanut 10,000 | 57.30 (39.34; 64.54) | 27.43 (6.66; 49.64) | **0.007** |
| Mean %CD63 Peanut 10-100 | 62.27 (35.72; 72.63) | 13.67 (5.32; 41.26) | **0.003** |
| AUC CD63 Peanut | 223.95 (131.91; 276.69) | 76.74 (31.88; 183.94) | **0.001** |
| Maximal %CD63+ to peanut | 70.31 (62.68; 80.26) | 31.91 (11.29; 58.14) | **0.001** |
| %CD63+ peanut/anti-IgE (%) | 1.34 (0.99; 1.45) | 0.64 (0.20; 1.21) | **0.010** |
| EC50 (ng/ml) - CD63 | 1.0 (1.0; 10.0) | 10.0 (1.0; 100.0) | 0.066 |
| CD-sens – CD63 | 51.27 (22.78; 110.65) | 9.32 (1.49; 53.74) | **0.014** |

**Table E6.** Correlations between severity (as measured by symptom score) and threshold (as measured by cumulative threshold dose) of allergic reactions to peanut and different diagnostic tests (n=44). Spearman correlation coefficient and p values are indicated. Significant correlations are highlighted in bold.

|  | **Symptom score** | **Cumulative threshold dose** |
| --- | --- | --- |
| Symptom score | - | r_s_= -0.067  p=0.645 |
| Time to reaction (minutes) | r_s_=0.040  p=0.790 | **r_s_=0.294**  **p=0.043** |
| SPT (mm) | **r_s_=0.417**  **p=0.003** | **r_s_= -0.394**  **p=0.005** |
| Peanut IgE specific activity (%) | **r_s_=0.398**  **p=0.008** | r_s_= -0.235  p=0.124 |
| Peanut-specific IgE (KU/L) | **r_s_=0.494**  **p<0.001** | **r_s_= -0.375**  **p=0.009** |
| Ara h 1-specific IgE (KU/L) | **r_s_=0.406**  **p=0.007** | r_s_= -0.310  p=0.043 |
| Ara h 2-specific IgE (KU/L) | **r_s_=0.536**  **p<0.001** | **r_s_= -0.420**  **p=0.005** |
| Ara h 3-specific IgE (KU/L) | r_s_=0.225  p=0.152 | r_s_= -0.302  p=0.052 |
| Ara h 8-specific IgE (KU/L) | r_s_=0.217  p=0.167 | r_s_= -0.051  p=0.749 |
| Ara h 9-specific IgE (KU/L) | r_s_=0.131  p=0.415 | r_s_=0.095  p=0.556 |
| Peanut-specific IgG4 (µg/ml) | r_s_=0.247  p=0.106 | r_s_=0.139  p=0.367 |
| Ratio peanut specific IgG4/IgE | r_s_= -0.276  p=0.070 | **r_s_=0.453**  **p=0.002** |
| %CD63+ peanut/anti-IgE (%) | **r_s_=0.548**  **p<0.001** | r_s_= -0.200  p=0.168 |
| CD-sens CD63 | **r_s_=0.391**  **p=0.008** | **r_s_= -0.518**  **p<0.001** |

**Supplementary figures:**

**Figure E1.** ROC curve and cut-offs for the use of CD63 peanut/anti-IgE for estimation of the severity [AUC (95%CI)=0.81 (0.68; 0.91)] of allergic reactions to peanut during challenges.

**Figure E2.** ROC curve and cut-offs for the use of CD-sens for the estimation of the threshold [AUC (95%CI)=0.75 (0.60; 0.87)] of allergic reactions to peanut during challenges.

**E-References:**

1. Ewan PW, Clark AT. Long-term prospective observational study of patients with peanut and nut allergy after participation in a management plan. Lancet 2001;357:111-5.
