# Supplementary material for: Distinct parameters of the basophil activation test reflect the severity and threshold of allergic reactions to peanut
Source: J Allergy Clin Immunol. 2015 Jan;135(1):179–86. doi: 10.1016/j.jaci.2014.09.001 (PMC4282725; doi:10.1016/j.jaci.2014.09.001)
Supplement: Figure E1 [file mmc2.pdf]

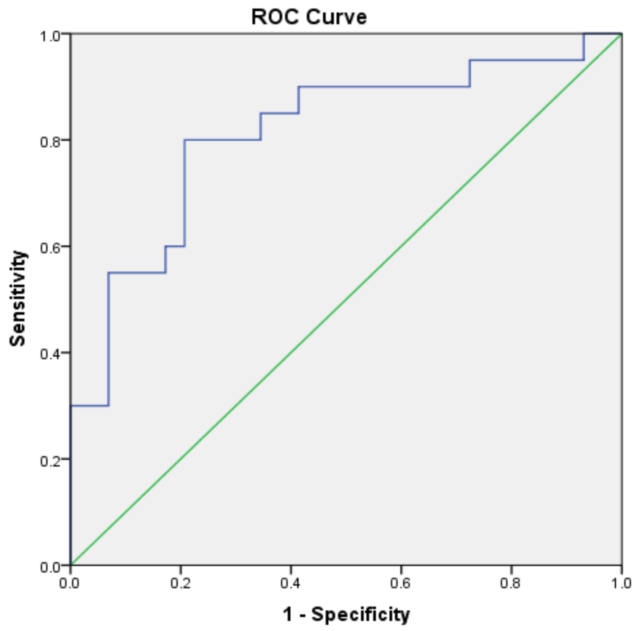

Cut-offs for CD63 peanut/anti-IgE	Sensitivity (95% CI)	Specificity (95% CI)	Positive predictive value (95% CI)	Negative predictive value (95% CI)
> 0.60	90.0 (68.3; 98.8)	58.6 (38.9; 76.5)	60.0 (40.6; 77.3)	89.5 (66.96; 98.7)
> 0.87	80.0 (56.3; 94.3)	79.3 (60.3; 92.0)	72.7 (49.8; 89.3)	85.2 (66.3; 95.8)
> 1.42	30.0 (11.9; 54.3)	96.6 (82.2; 99.9)	85.7 (42.1; 99.6)	66.7 (50.5; 80.4)
