# Supplementary material for: Distinct parameters of the basophil activation test reflect the severity and threshold of allergic reactions to peanut
Source: J Allergy Clin Immunol. 2015 Jan;135(1):179–86. doi: 10.1016/j.jaci.2014.09.001 (PMC4282725; doi:10.1016/j.jaci.2014.09.001)
Supplement: Figure E2 [file mmc3.pdf]

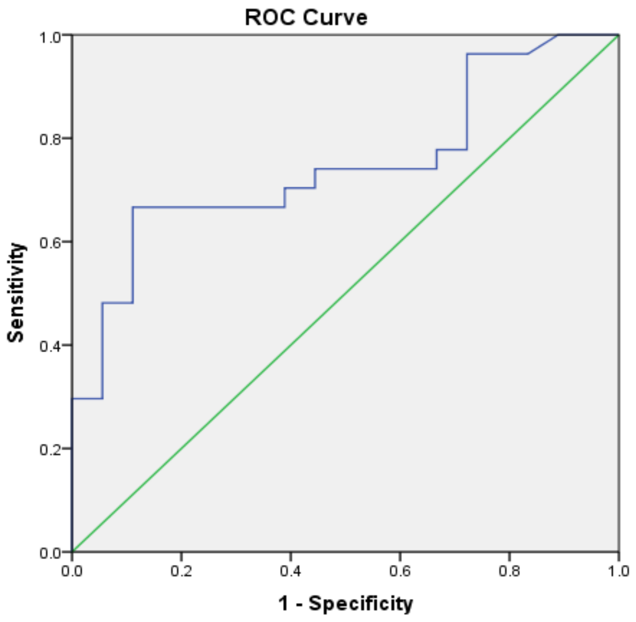

Cut-offs for CD-sens	Sensitivity (95% CI)	Specificity (95% CI)	Positive predictive value (95% CI)	Negative predictive value (95% CI)
> 0.9	96.3 (81.0; 99.9)	27.8 (9.1; 53.5)	66.7 (49.8; 80.9)	83.3 (35.9; 99.6)
> 16.5	66.7 (46.0; 83.5)	88.9 (65.3; 98.6)	90.0 (68.3; 98.8)	64.0 (42.5; 82.0)
> 104.7	29.6 (13.8; 52.2)	100.0 (81.5; 100.0)	100.0 (63.1; 100.0)	48.6 (31.9; 65.6)
